# The Construction and Analysis of ceRNA Network and Patterns of Immune Infiltration in Colon Adenocarcinoma Metastasis

**DOI:** 10.3389/fcell.2020.00688

**Published:** 2020-08-04

**Authors:** Zhengyan Chang, Runzhi Huang, Wanting Fu, Jiehan Li, Guo Ji, Jinglei Huang, Weijun Shi, Huabin Yin, Weifeng Wang, Tong Meng, Zongqiang Huang, Qing Wei, Huanlong Qin

**Affiliations:** ^1^Department of Pathology, Shanghai Tenth People’s Hospital, Tongji University School of Medicine, Shanghai, China; ^2^Department of Orthopaedics, The First Affiliated Hospital of Zhengzhou University, Zhengzhou, China; ^3^Division of Spine, Department of Orthopedics, Tongji Hospital Affiliated to Tongji University School of Medicine, Shanghai, China; ^4^Tongji University School of Medicine, Tongji University, Shanghai, China; ^5^Department of Orthopedics, Shanghai General Hospital, School of Medicine, Shanghai Jiao Tong University, Shanghai, China; ^6^Department of Central Laboratory, Shanghai Tenth People’s Hospital, Shanghai, China; ^7^Tongji University Cancer Center, Shanghai Tenth People’s Hospital of Tongji University, School of Medicine, Tongji University, Shanghai, China; ^8^Department of Gastrointestinal Surgery, Shanghai Tenth People’s Hospital, Tongji University School of Medicine, Shanghai, China

**Keywords:** colon adenocarcinoma, metastasis, prognosis, competing endogenous RNA network, tumor-infiltrating immune cell

## Abstract

**Background:**

Colon adenocarcinoma (COAD) is a malignant and lethal tumor in digestive system and distance metastasis lead to poor prognosis. The metastasis-specific ceRNAs (competitive endogenous RNAs) and tumor-infiltrating immune cells might associate with tumor prognosis and distance metastasis. Nonetheless, few studies have concentrated on ceRNAs and Immune cells in COAD.

**Methods:**

The gene expression profile and clinical information of COAD were downloaded from TCGA and divided into two groups: primary tumors with or without distance metastasis. We applied comprehensive bioinformatics methods to analyze differential expression genes (DEGs) related to metastasis and establish the ceRNA networks. The Cox analysis and Lasso regression were utilized to screen the pivotal genes and prevent overfitting. Based on them, the prognosis prediction nomograms were established. The cell type identification by estimating relative subsets of RNA transcripts (CIBERSORT) algorithm was then applied to screen significant tumor immune-infiltrating cells associated with COAD metastasis and established another prognosis prediction model. Ultimately, co-expression analysis was applied to explore the relationship between key genes in ceRNA networks and significant immune cells. Multiple databases and preliminary clinical specimen validation were used to test the expressions of key biomarkers at the cellular and tissue levels.

**Results:**

We explored 1 significantly differentially expressed lncRNA, 1 significantly differentially expressed miRNA, 8 survival-related immune-infiltrating cells, 5 immune cells associated with distance metastasis. Besides, 3 pairs of important biomarkers associated with COAD metastasis were also identified: T cells follicular helper and hsa-miR-125b-5p (*R* = −0.200, *P* < 0.001), Macrophages M0 and hsa-miR-125b-5p (*R* = 0.170, *P* < 0.001) and Macrophages M0 and FAS (*R* = −0.370, *P* < 0.001). Multidimensional validation and preliminary clinical specimen validation also supported the results.

**Conclusion:**

In this research, we found some significant ceRNAs (FAS and hsa-miR-125b-5p) and tumor-infiltrating immune cells (T cells follicular helper and Macrophages M0) might related to distance metastasis and prognosis of COAD. The nomograms could assist scientific and medical researchers in clinical management.

## Introduction

Colon adenocarcinoma (COAD) ranks the third and fourth place in the rankings of cancer incidence and mortality all over the world, respectively (Mayer, 1991; Haggar and Boushey, 2009). More than one million new COAD cases and about COAD-related 700,000 death occur in every year (Haggar and Boushey, 2009). Although surgery, chemotherapy and target therapy have increased the overall survival of patients with COAD significantly, around 50% of COAD patients experience distant metastasis which is also the most frequent cause of treatment failure (Stein et al., 2009; Tang et al., 2016). Once the tumor metastasis occurs, 5-year-survival rate is significantly reduced to 8.1% (O’connell et al., 2004). Due to the insufficient studies on COAD metastasis, the mechanism of COAD metastasis is still unclear and the predicting biomarkers are mainly focused on overall survival (Beahrs, 1992; Chatterjee and Zetter, 2005; Xing et al., 2006; Duffy et al., 2007). Thus, it is an urgent need to probe the mechanism of COAD metastasis and identify its predictors.

The ceRNA network postulates that any RNA transcript harboring microRNA (miRNA)-response elements can sequester miRNAs from other targets, thereby regulating their expression and coordinating biological processes (Karreth and Pandolfi, 2013). Previous studies have focused on this point and revealed that the ceRNA network took part in colon cancer growth, metastasis and prognosis, such as hsa-circ-000984 and miR-145 (Zhou et al., 2016; Xu et al., 2017). In addition, the existence of tumor-infiltrating immune cells, especially leukocytes, is also an essential evident witnesses of the activity of immune system and is supposed to play critical roles in cancer growth, progression and metastasis including the COAD (Dadabayev et al., 2004; Halama et al., 2009; Palmqvist et al., 2013). However, there are only a few studies focusing on the regulatory mechanism between ceRNA networks and tumor-infiltrating immune cells, not containing COAD (Lou et al., 2019; Yang et al., 2019; Zhao R. et al., 2019).

In the present study, we set up a ceRNA network based on COAD metastasis related ceRNAs, which were identified by gene expression profiling from the TCGA database. In addition, we evaluated the ratios of tumor-infiltrating immune cells associated with COAD metastasis by using the “cell type identification by estimating relative subsets of RNA transcripts (CIBERSORT)” algorithm. Depended on the differentially expressed immune cells and ceRNAs types, nomograms were constructed to predict COAD metastasis and prognosis. Furthermore, the correlation analysis was performed to explore the metastasis mechanism of COAD. The constructed nomograms and the identified regulatory mechanism might provide new insights for the prediction and treatment of COAD metastasis in clinic.

## Materials and Methods

### Data Selection and Analysis of Differential Gene Expression

This study was endorsed by the Ethics Committee of the Shanghai Tenth People’s Hospital affiliated to Tongji University. From the cancer genome atlas (TCGA^1^) database, RNA profiles including HTseq-count and fragments per kilobase of exon per million reads mapped (FPKM) of 459 primary COADs were downloaded. The experimental group and the control group in this study were 91 primary COADs with distant metastasis and 368 primary COADs without distant metastasis. We also retrieved the demographic information (age, gender, race and so on), survival endpoint (vital status, days to death and days to last follow-up) and stage and histological type of primary and new tumors of each patient. When non-colon cancer specific expression genes (The expression of these genes was not detected in both the control group and experimental group) had been filtered, the edgeR method was utilized to identify differentially expressed RNAs including lncRNAs, miRNAs, and mRNAs. The downregulated or upregulated gene was, respectively, defined with a false discovery rate (FDR) *P* value < 0.05, the log(fold-change) > 1.0 or < −1.0.

### Construction of the ceRNA Network

Ahead of analyzing the basic statistics, download information which was based on experimental verification from miRTarBase (Chou et al., 2018)^2^ about the miRNA–mRNA inter-action and downloaded the lncRNA–miRNA interaction information from lncbase v.2 Experimental Module (Paraskevopoulou et al., 2016)^3^ at the same time. All interactions are verified by molecular mechanisms of direct mechanisms (e.g., luciferase reporter assays and co-immunoprecipitation). After that, prominent results shown in hypergeometric testing and correlation analysis by miRNAs which regulated both lncRNAs and mRNAs were singled out to construct the ceRNA network with Cytoscape v.3.5.1 (Shannon et al., 2003).

### Survival Analysis and Nomograms of Pivotal Members in the ceRNA Network

Firstly, all components of the ceRNA network were incorporated into the Cox model and in order to assure multifaceted models were not overfitting, the Lasso regression was used. In order to identify the prognostic value of all biomarkers, Cox proportional hazards model was applied. Then, a nomogram, based on the multivariable models, was built to predict the survival of COAD patients. To estimate the accuracy and discrimination of the nomogram, calibration curves and receiver operating characteristic curves (ROC) were applied. Finally, Kaplan–Meier survival analysis was used to validate each biomarker.

### CIBERSORT Estimation

The CIBERSORT algorithm was utilized to assess the 22 kinds of immune cell types in carcinoma COAD. Only if those samples which had a CIBERSORT output of *P* < 0.05 were deemed to be worthy of further analysis. It had significant differences in the pro-portion between primary tumors with and without distance metastasis by finding the immune-infiltrating cells with the Wilcoxon rank-sum test.

### Survival Analysis and Nomograms of Pivotal Members for the Tumor Immune Cells

In order to render certain that the multifactor models weren’t overfitting, we integrated all significant cells into the Cox model and then the reduced Cox model was constructed. Applying for Cox proportional hazards model and Kaplan–Meier survival analysis to identify the prognostic value of all tumor-infiltrating immune cells. Then, the calibration curves and receiver operating characteristic curves (ROC) were performed to estimate the accuracy and discrimination of the nomogram. Ultimately, based on Pearson correlation analysis, a co-expressed heatmap was drawn to show the correlation of various immune cells.

### Multidimensional Validation

To minimize bias, multiple databases which included Oncomine (Rhodes et al., 2004), The Human Protein Atlas (Uhlen et al., 2015), Gene Expression Profiling Interactive Analysis (GEPIA) (Tang et al., 2017), LinkedOmics (Vasaikar et al., 2018), String (Szklarczyk et al., 2011), Lncrna2target V2.0 (Cheng et al., 2019), exoRBase (Li S. et al., 2018) and PROGeneV2 (Goswami and Nakshatri, 2014) were utilized to decent gene and protein expression levels of key biomarkers at the tissue and cellular levels. And the marker genes of the co-expressed immune cells were selected from CellMarker database.

### Immunohistochemistry (IHC) and Quantitative Reverse Transcriptase-PCR (qRT-PCR)

Four-micron sections of formalin-fixed paraffin-embedded (FFPE) tissue blocks from 20 primary COAD samples, including ten diagnosed with metastasis and ten diagnosed without metastasis, were deparaffinized and dehydrated. Following routine rehydration, antigen retrieval, and blocking procedures, the sections were incubated overnight with FAS antibody (1:50 dilution, Proteintech), NES antibody (1:50 dilution, Proteintech), CD68 antibody (1:50 dilution, Proteintech), CD3 antibody (1:50 dilution, Proteintech) and BCL6 antibody (1:50 dilution, Proteintech) at 4°C, respectively. Next, all slides were ladeled polymer HRP for 30 min and hematoxylin as a counterstain for 5 min at room temperature. Two pathologists read the pathological sections and determined positive results when the cytoplasm of cancer cells was stained. The percentage scoring of tumor cells was as follows: negative (0), yellowish (1–4), light brown (5–8), and dark brown (9–12). Negative controls had the primary antibody replaced by buffer. Additionally, the non-parametric test and correlation analysis were performed to evaluate the relationship between the percentage scoring of tumor cells and clinical features of patients.

Quantitative reverse transcriptase-PCR (qRT-PCR) was used to quantitative expression of key miRNAs in the ceRNA network. According to the manufacturer’s instructions, total RNA was extracted from the tissues using TRIzol Reagent (Invitrogen). The OD260/OD280 ratio of total RNA extracted from tissue samples ranged from 1.8 to 2.0. Reverse transcription was performed in a 10 μl reaction volume using M-MLV reverse transcriptase (Takara, Japan) with 1 μg of RNA. Quantitative real-time PCR was performed using an ABI 7900 Detection System with the SYBR Premix Ex Taq^TM^ (Takara, Japan). Amplification included an initial denaturation step for 30 s at 95°C, followed by 40 cycles of PCR at 95°C for 5 s, and at 60°C for 31 s and a dissociation stage for 15 s at 95°C, 60 s at 60°C and 15 s at 95°C. After the reactions were complete, the cycle threshold (CT) data were determined using fixed threshold settings and the mean CT was determined from triplicate PCRs. A comparative CT method was used to compare each condition to the control reactions. mRNA levels were normalized to U6. The relative amount of gene normalized to control was calculated with the equation 2^–ΔΔCT^.

### Data Analysis

Only two-sided *P* value < 0.05 was considered to have necessary to statistic. All statistical analysis was put into effect with R version 3.5.1 software (Institute for Statistics and Mathematics, Vienna, Austria; www.r-project.org) (Package: GDCRNATools (Li R. et al., 2018), edgeR, ggplot2, rms, glmnet, preprocessCore, survminer, timeROC).

## Results

### Identification of the Markedly Different Expressed Genes

**FIGURE 1:**
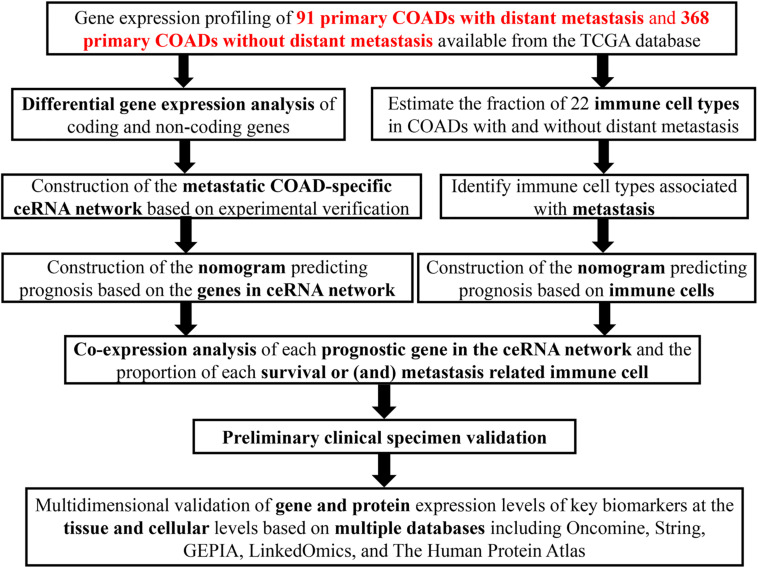
The flow chart of the whole research process.

**FIGURE 2:**
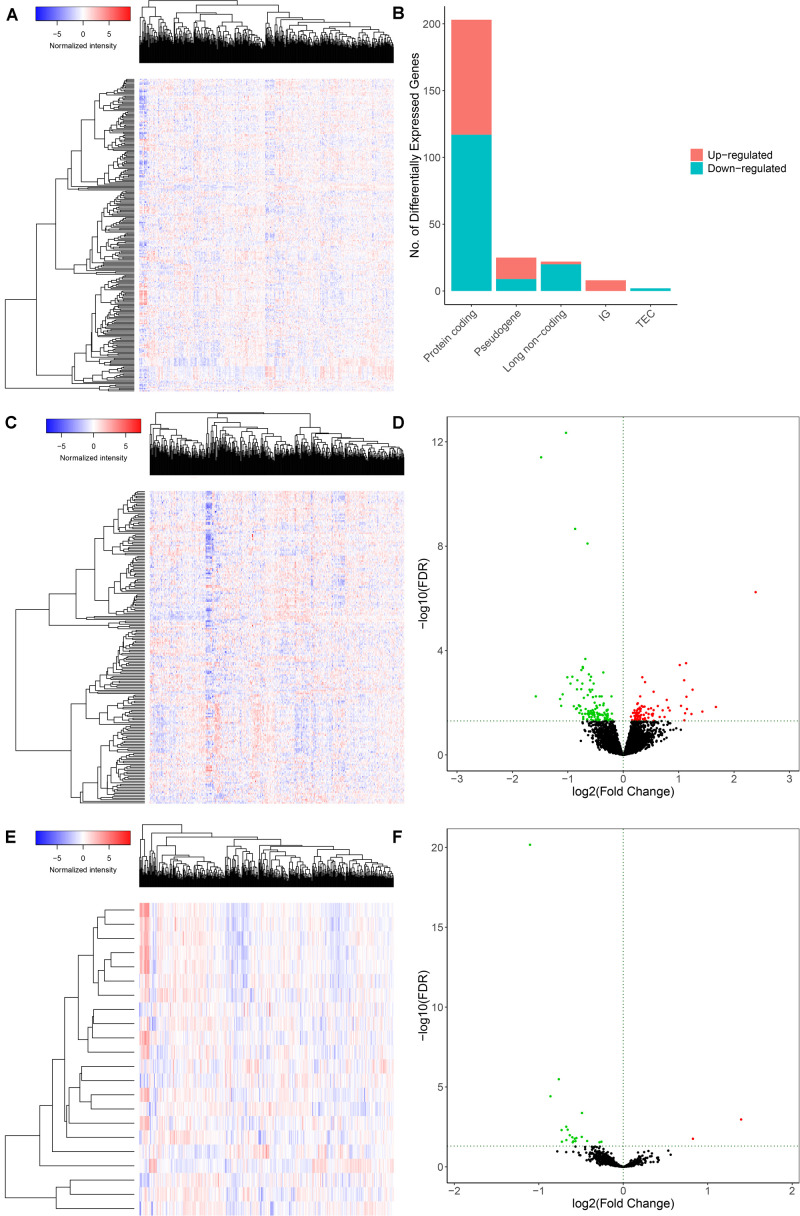
Genes and lncRNAs which were significant differentially expressed between primary and distance metastasis colon carcinoma. The cutoffs which we set was log(fold-change) > 1.0 or < –1.0 and FDR < 0.05 and then we identified 260 differentially expressed genes **(A,B)** which include 203 protein-coding genes (117 downregulated and 86 upregulated) **(C,D)** and 22 lncRNAs (21 downregulated and 1 upregulated) **(E,F)** in the total of 14,447 lncRNAs, 2,588 miRNAs and 19,660 mRNAs from the TCGA database. TCGA: the cancer genome atlas.

The schematic diagram was constructed to display the methods and results of our study (Figure 1). A total of 14,447 lncRNAs, 2,588 miRNAs and 19,660 mRNAs were identified from the TCGA database of COAD (Figures 2A,B). Using the log(fold-change) > 1.0 or < −1.0 and FDR < 0.05 as the cutoffs, a total of 203 protein-coding genes (117 downregulated and 86 upregulated) (Figures 2C,D), 22 lncRNAs (21 downregulated and 1 upregulated) (Figures 2E,F) and 18 miRNAs (9 downregulated and 9 upregulated) were differently expressed between the colon carcinoma patients with and without distant metastasis.

### The Constructed ceRNA Network and the Survival Analysis

**FIGURE 3:**
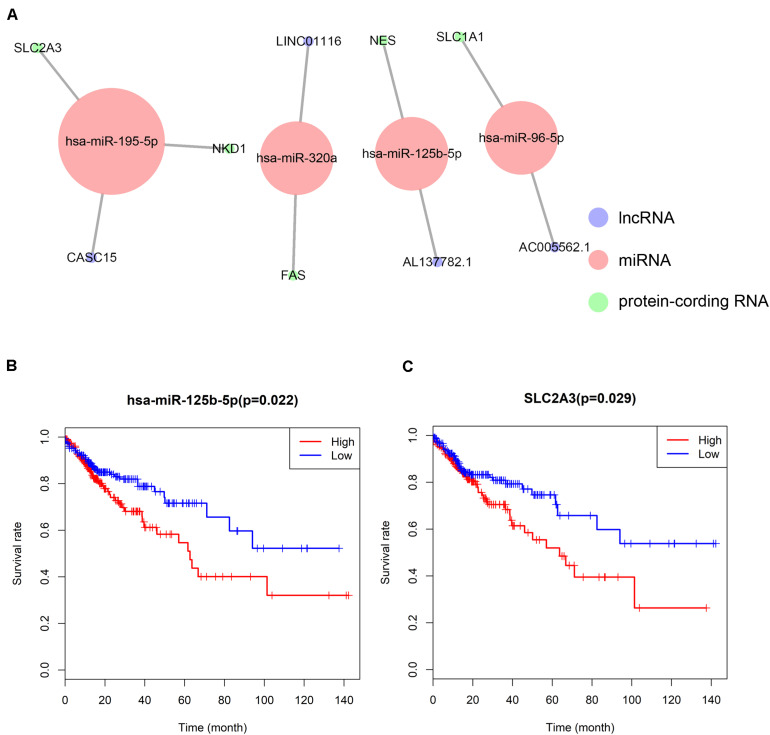
The ceRNA network constructed for distance metastasis colon carcinoma **(A)** and the Kaplan–Meier survival curves of the important members of the ceRNA network **(B,C)**.

**FIGURE 4:**
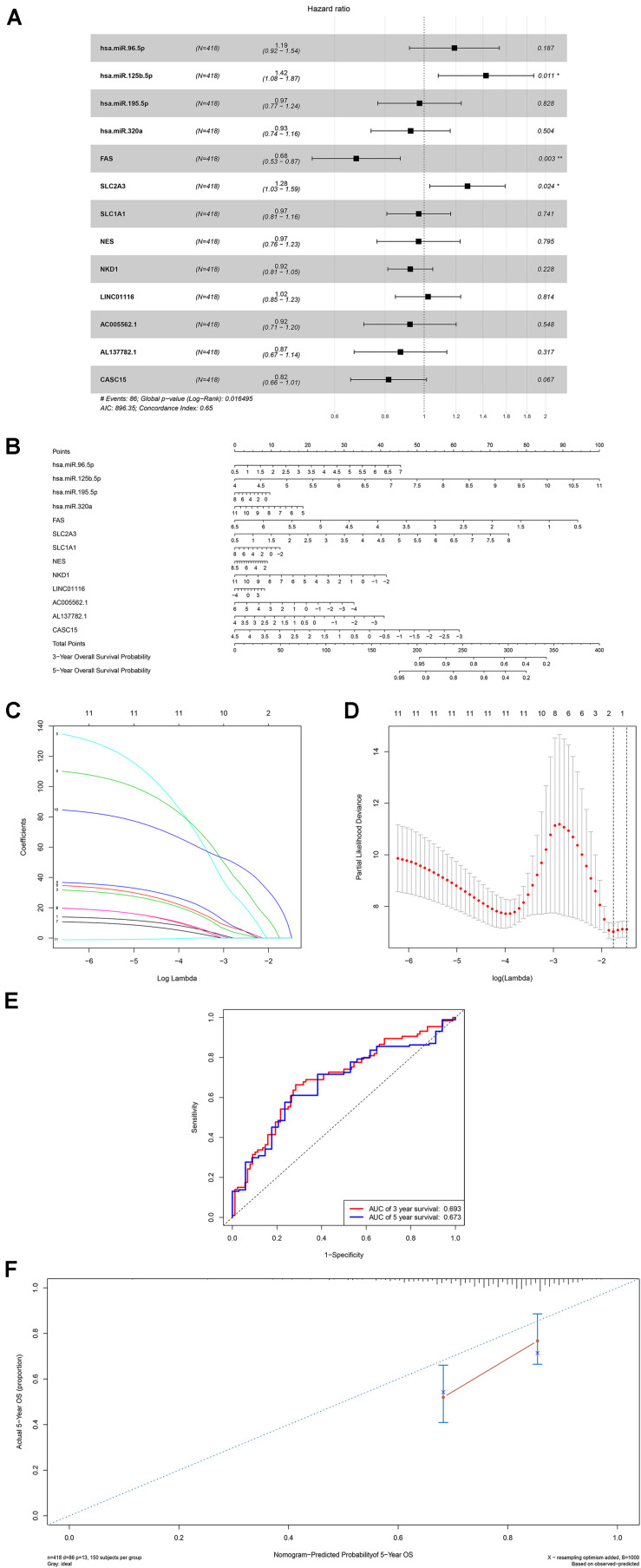
The results of the multivariate Cox regression **(A)**, nomogram **(B)** and model diagnosis process Lasso regression and ROC curves **(C–F)** which were constructed by the significant members of the ceRNA network. ROC, receiver operating characteristic curves. *P < 0.05.

A ceRNA network was constructed composing of 13 genes (4 lncRNAs, 4 miRNAs and 5 mRNAs). All these genes made up 4 lncRNA–miRNA pairs and 5 miRNA–mRNA pairs (Figure 3A and Table 1). In the Cox regression and Kaplan–Meier method which were applied to test the pertinence of the colon cancer’s biomarkers for the prognosis, only two significant genes were identified among the ceRNA network (SLC2A3, *P* = 0.029; hsa-miR-125b-5p, *P* = 0.022) (Figures 3B,C). All thirteen genes in the ceRNA network were integrated into a Cox proportional hazards model to evaluate their prognostic values (Figure 4A). Based on the model, a nomogram was established to predict the 3-year and 5-year overall survival probability of COAD patients (Figure 4B). The Lasso regression was used to test the signification of these genes for the modeling (Figures 4C,D). Both the ROC and the calibration curves (Figures 4E,F) were applied and manifested an acceptable accuracy (Area Under Curve (AUC) of 3-year survival: 0.693; AUC of 5-year survival: 0.673) and calibration of the nomogram.

**TABLE 1:** Hypergeometric testing and correlation analysis results of ceRNAs network.

LncRNA	Protein-coding RNA	MiRNAs	Correlation P	Hypergeometric test P
AC005562.1	SLC1A1	hsa-miR-96-5p	0.0104300454151637	0.0280898876404494
AL137782.1	NES	hsa-miR-125b-5p	0.0395689952024311	0.0407867741837382
LINC01116	FAS	hsa-miR-320a	0.00298820801951597	0.0302446669455895
CASC15	SLC2A3	hsa-miR-195-5p	2.92413632053211E-09	0.0223526026570475
CASC15	NKD1	hsa-miR-195-5p	9.1639176614199E-14	0.0442327365989891

*ceRNAs, competing endogenous RNAs; LncRNA, long non-coding RNA; MiRNA, microRNA.*

### The Composition of Tumor-Infiltrating Immune Cells Between Metastatic and Non-metastatic Primary COAD

**FIGURE 5:**
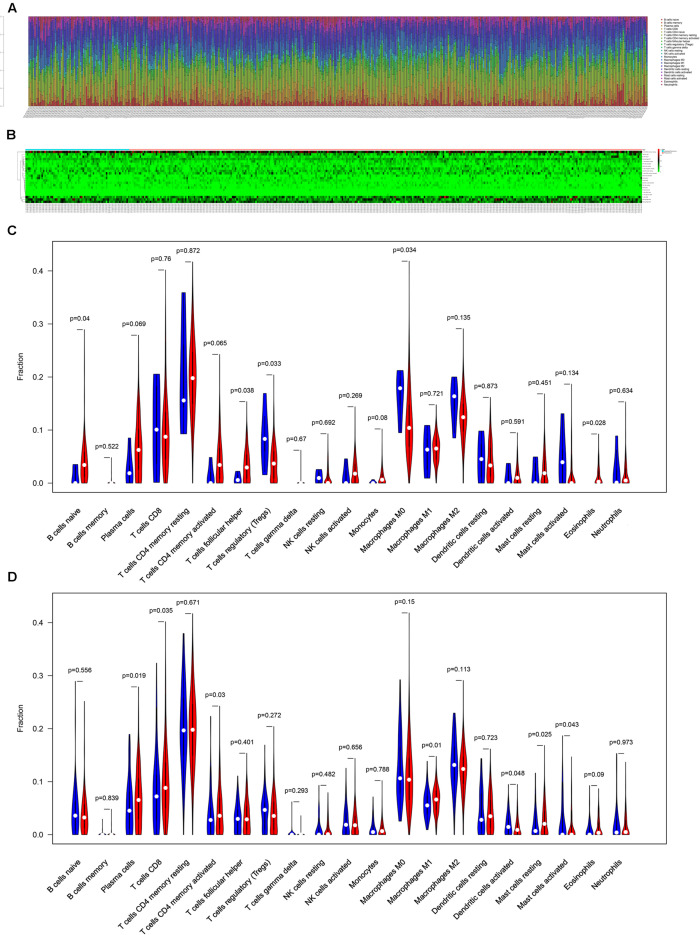
The composition of immune cells assessed by CIBERSORT algorithm in colon cancer **(A,B)** and the identification of prominent tumor-infiltrating immune cells related to the distance metastasis of colon cancer **(C)** and associated with the metastasis and recurrence of it **(D)**. CIBERSORT: cell type identification by estimating relative subsets of RNA transcripts.

The composition of significant tumor-infiltrating immune cells was assessed by CIBERSORT algorithm in the COAD tissue. The histogram (Figure 5A) and the heat map (Figure 5B) could indicate that T cells CD4 memory resting, T cells CD8, Macrophages M0 and Macrophages M2 were obviously tested high-expression in primary tumor and distance metastasis tissues, and they might play essential roles in colon cancer. Moreover, the Wilcoxon rank-sum test suggested that T cells follicular helper (*P* = 0.038), B cells naive (*P* = 0.040), Tregs (*P* = 0.033), Macrophages M0 (*P* = 0.034) and Eosinophils (*P* = 0.028) had significant differences in the immune cell fractions between distance metastasis and primary colon adenocarcinoma (Figure 5C) and T cells CD4 memory activated (*P* = 0.030), Plasma cells (*P* = 0.019), T cells CD8 (*P* = 0.035), Dendritic cells activated (*P* = 0.048), Dendritic cells resting (*P* = 0.048), Macrophages M1 (*P* = 0.010), Mast cells resting (*P* = 0.043), and Mast cells activated (*P* = 0.025) had obvious differences in the immune cell fractions between distance metastasis and the recurrence of COAD (Figure 5D).

### The Composite and Co-expression Analysis About Genes and Immune Cells for Prognosis and Distance Metastasis

**FIGURE 6:**
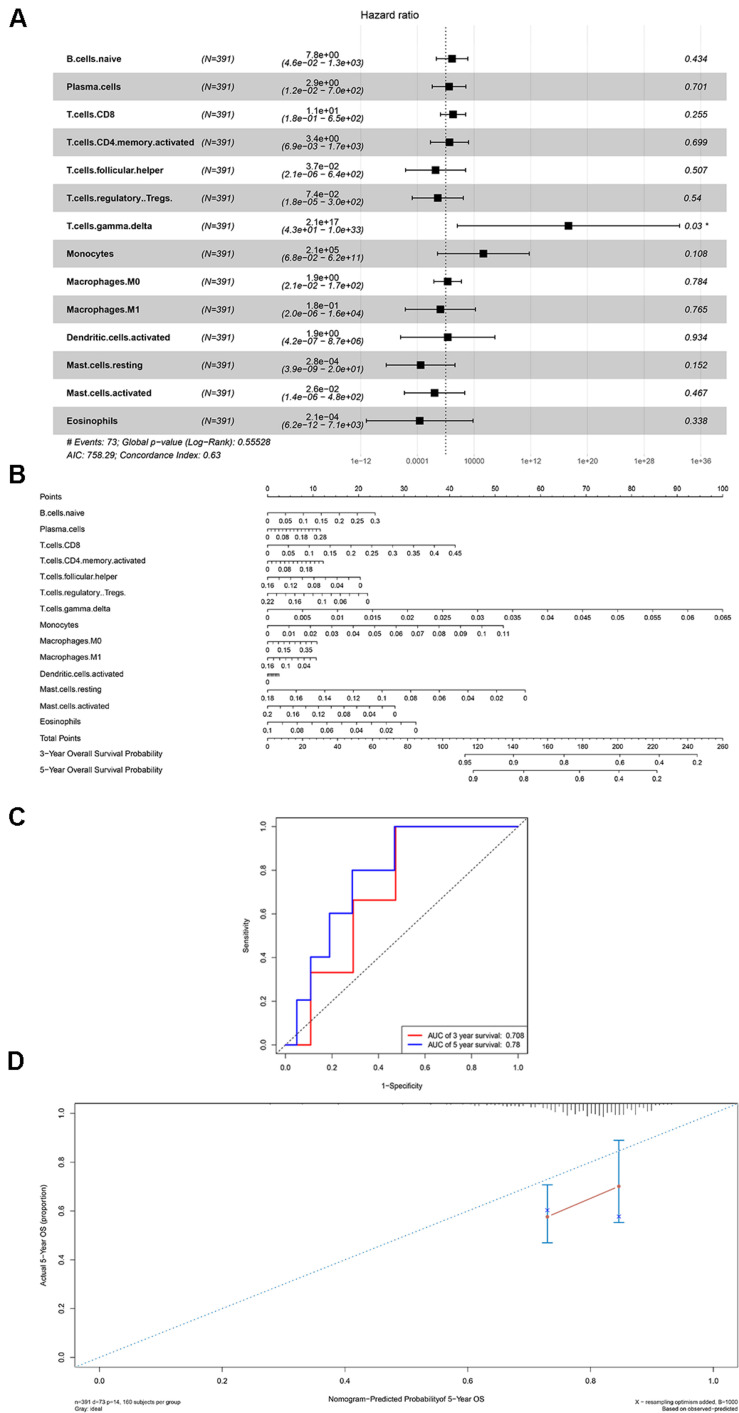
The results of the multivariate Cox regression **(A)**, nomogram **(B)** and model diagnosis process ROC and calibration curves **(C,D)** on account of the significant immune cells which were associated with survival. *P < 0.05.

**FIGURE 7:**
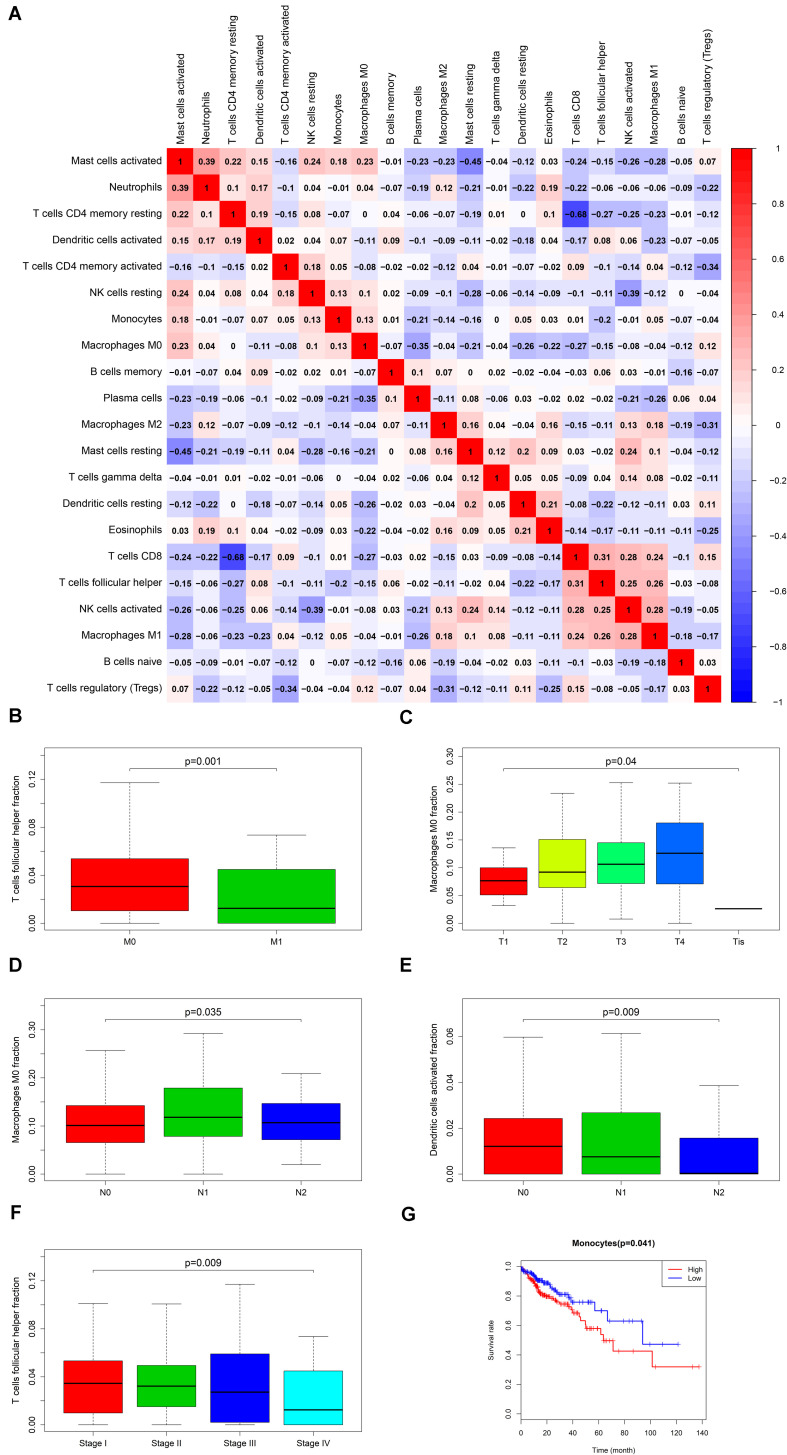
The result of the co-expression analysis between significant immune cells **(A)** and the box plots about TMN stages of T cells follicular helper; Macrophages M0 and Dendritic cells activated **(B–F)**, and the result of the Kaplan-Meier curves of Monocyte **(G)**.

**FIGURE 8:**
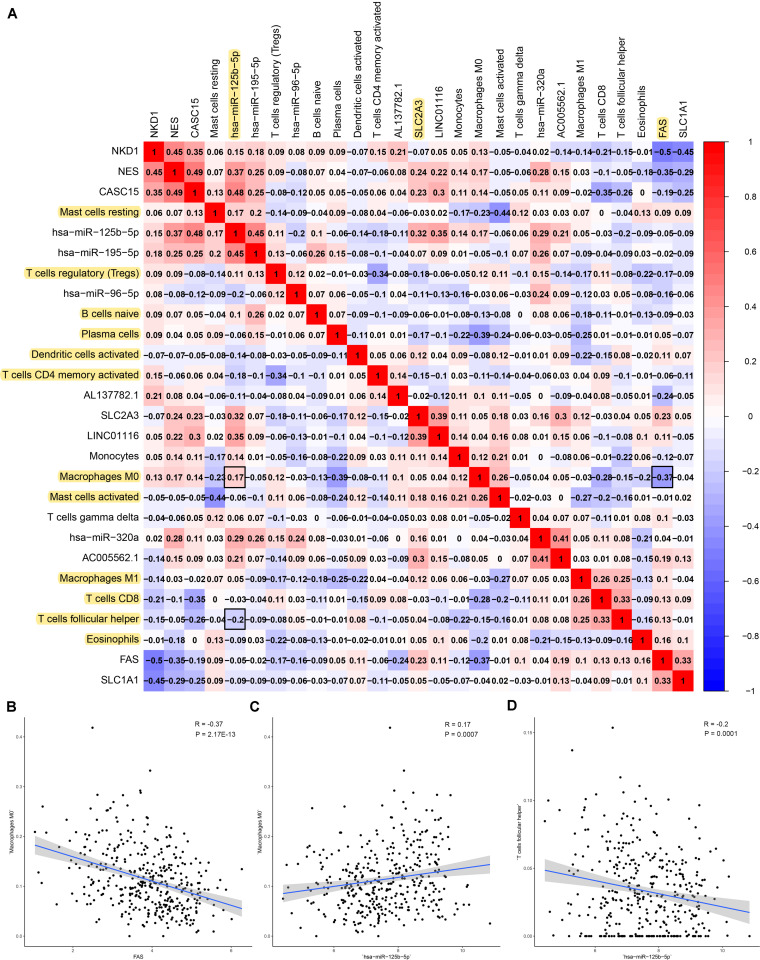
The result of the co-expression analysis between tumor-infiltrating immune cells and key members of ceRNA network. The co-expression heatmap illustrated some significant co-expression patterns about key members in the ceRNA network and key members in the immune cells **(A)**. T cells follicular helper and hsa-miR-125b-5p (*R* = −0.200, *P* < 0.001) Macrophages M0 and hsa-miR-125b-5p (*R* = 0.170, *P* < 0.001) and Macrophages M0 and FAS (*R* = −0.370, *P* < 0.001) and these could describe the significant relationship between the key biomarkers and distance metastasis COAD **(B–D)**.

Firstly, an initial Cox regression model with all significant tumor immune cells was constructed (Figure 6A). As the models of genes, basing on the multivariable model, a nomogram was utilized to show 14 kinds of vital immune cells affections to 3-year and 5-year overall survival probability (Figure 6B). Besides, the ROC and the calibration curve demonstrated could show the nomogram’s discrimination and concordance (AUC of 3-year survival: 0.708; AUC of 5-year survival: 0.780) (Figures 6C,D). And Kaplan–Meier survival curve also suggested significant prognostic value of the risk score generated from the multivariable model (*P* < 0.001) (Supplementary Figure S1). The co-expression analysis using prognostic tumor-infiltrating immune cells in primary COAD samples with and without distant metastasis was performed (Figure 7A). T cells follicular helper (M stage: *P* = 0.001; Staging: *P* = 0.009), Macrophages M0 fraction (T stage: *P* = 0.035; N stage: *P* = 0.040), dendritic cells activated (N stage: *P* = 0.009) and monocyte (Kaplan–Meier survival analysis: *P* = 0.041) all showed significant correlation with clinic features (Figures 7B–G). Finally, Figure 8 illustrated some significant co-expression patterns about key members in the ceRNA network and key members in the immune cells. T cells follicular helper and hsa-miR-125b-5p (*R* = −0.200, *P* < 0.001) Macrophages M0 and hsa-miR-125b-5p (*R* = 0.170, *P* < 0.001) and Macrophages M0 and FAS (*R* = −0.370, *P* < 0.001) and these could describe the significant relationship between the key biomarkers and distance metastasis COAD.

### Preliminary Clinical Specimen Validation

**FIGURE 9:**
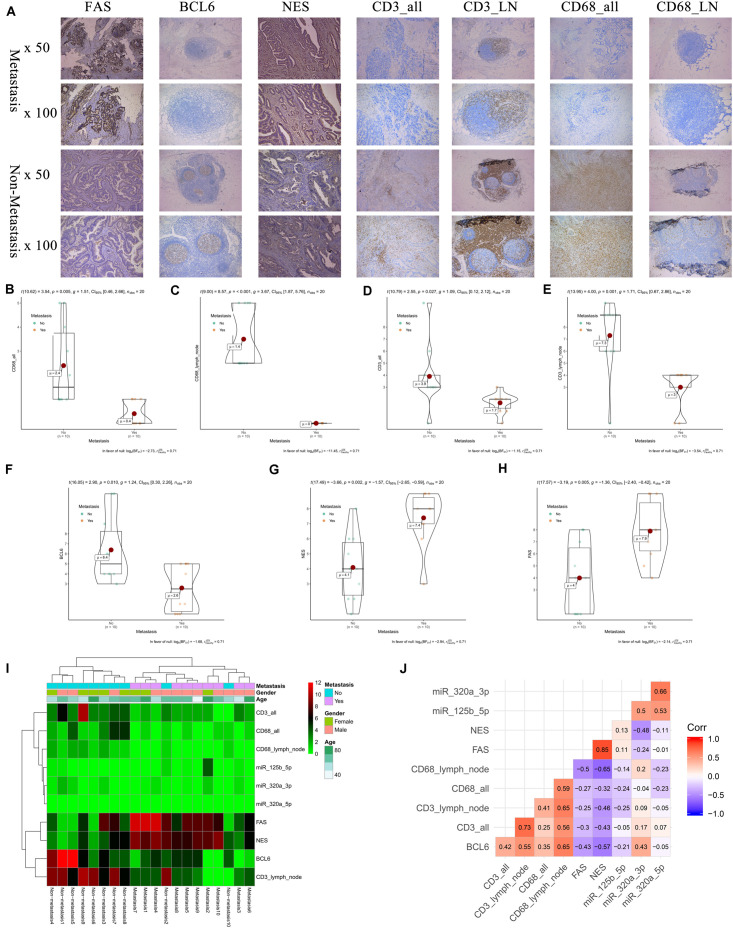
The results of preliminary clinical specimen validation. The results of IHC (20 primary COAD samples including 10 with metastasis and 10 without metastasis) suggested that proteins of FAS, NES, CD3, CD68, and BCL6 were significantly different in COADs diagnosed with metastasis and without metastasis **(A)**. The results of non-parametric tests also confirmed that these differences were statistically significant (CD68 in all tissue, *P* = 0.005; CD68 in lymph nodes, *P* < 0.001; CD3 in all tissue, *P* = 0.027; CD3 in lymph nodes, *P* = 0.001; BCL6, *P* = 0.010; NES, *P* = 0.002; FAS, *P* = 0.005) **(B–H)**. The expression levels of FAS, NES, CD3, CD68, BCL6 and key miRNAs (miR-320a-3p, miR-320a-5p, and miR-125b-5p) in the ceRNA network were illustrated in the heatmap **(I)**. Additionally, despite the limitation to the sample size, Additionally, despite the limitation to the sample size, CD68 (Marker of Macrophages M0) and FAS (*R* = –0.500), CD68 and NES (*R* = –0.650) shown co-expression trends **(J)**.

The results of IHC (20 primary COAD samples including 10 with metastasis and 10 without metastasis) suggested that proteins of FAS, NES, CD3, CD68, and BCL6 were significantly different in COADs diagnosed with metastasis and without metastasis (Figure 9A). The results of non-parametric tests also confirmed that these differences were statistically significant (CD68 in all tissue, *P* = 0.005; CD68 in lymph nodes, *P* < 0.001; CD3 in all tissue, *P* = 0.027; CD3 in lymph nodes, *P* = 0.001; BCL6, *P* = 0.010; NES, *P* = 0.002; FAS, *P* = 0.005) (Figures 9B–H). The expression levels of FAS, NES, CD3, CD68, BCL6 and key miRNAs (miR-320a-3p, miR-320a-5p, and miR-125b-5p) in the ceRNA network were illustrated in the heatmap (Figure 9I). Additionally, despite the limitation to the sample size, CD68 (Marker of Macrophages M0) and FAS (*R* = −0.500), CD68 and NES (*R* = −0.650) shown co-expression trends (Figure 9J).

### Multidimensional Validation

**FIGURE 10:**
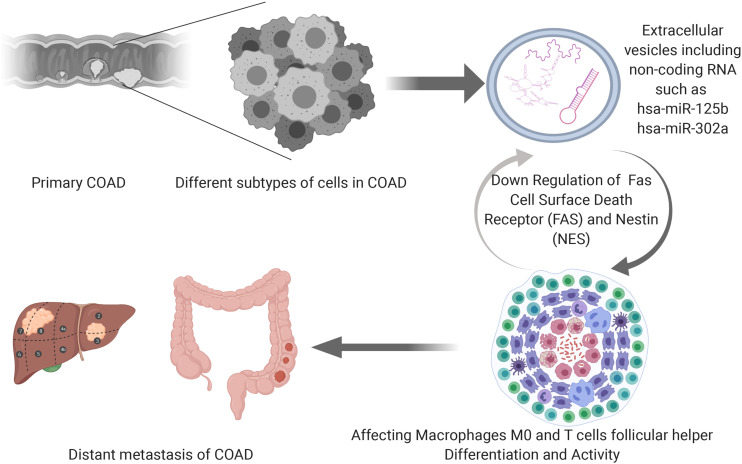
The speculative mechanism diagram containing key ceRNAs and immune cells.

The speculative mechanism diagram containing key ceRNAs and immune cells were summarized in Figure 10. And to explore the significant gene and protein expressions of significant ceRNAs and immune cells, surface markers of key immune cells were retrieved from CellMarker database and a dimensional verification of multiple databases were performed to the primary COAD, normal colon and cancer cell lines. FAS and T cells follicular helper are the significant ceRNA and tumor-infiltrating immune cell. BCL6, CD3, MME, CXCL13, and ICOS were top five most reported markers of T cells follicular helper (28). Firstly, across 9 studies, FAS (Median rank 347.0, *P* = 4.98E-6) was lowly expressed in primary COAD compared to normal colon and BCL6 (Median rank 6472.0, *P* = 0.014), CXCL13 (Median rank 406.0, *P* = 1.41E-8), CD3E (Median rank 109.0, *P* = 1.81E-6) and MME (Median rank 6102.5, *P* = 0.027) showed obvious difference between primary COAD and normal colon in the Oncomine (Supplementary Figure S2). At the transcriptome level, the correlation of FAS, CXCL13 (*P* < 0.001, *R* = 0.400) and BCL6 (*P* < 0.001, *R* = 0.340) was significantly different between COAD and normal colon in GEPIA (Supplementary Figure S3). LinkedOmics database results showed that FAS (*P* = 4.623E-6) and hsa-miR-125b-5p (*P* = 1.102E-9) were significantly related to tumor purity and tumor stage of COAD (Supplementary Figure S4). Besides, Pearson-correlation analysis also drew significant results between EGFR and CXCL13 (*P* = 4.536E-5), CD3D (*P* = 1.511E-13) and CD3E (*P* = 4.536E-5) in LinkedOmics (Supplementary Figure S4). What’s more, the results of data mining of The Human Protein Atlas indicated that the detection of protein FAS, CD3G and MME were significantly different in COAD and normal colon (Supplementary Figure S5). The results of String database showed the protein-protein interaction network of FAS, CD3D, and CD3E (Supplementary Figure S6). In addition, Supplementary Figure S7 illustrated the base sequences and duplex structures of miR-320a and miR-125b-5p available from the MirTarBase. Furthermore, the scientific hypothesis of this study was inspired by some basic experimental studies, which reported that exosomes secreted by tumor cells contain ceRNAs, exosomes act on immune cells, and ultimately mediate phenotypes. Therefore, the expression levels of key lncRNA (LINC01116) and mRNAs (FAS and NES) were validated in the RNA-seq data of exosomes (Supplementary Figure S8) by exoRBase.

**FIGURE 11:**
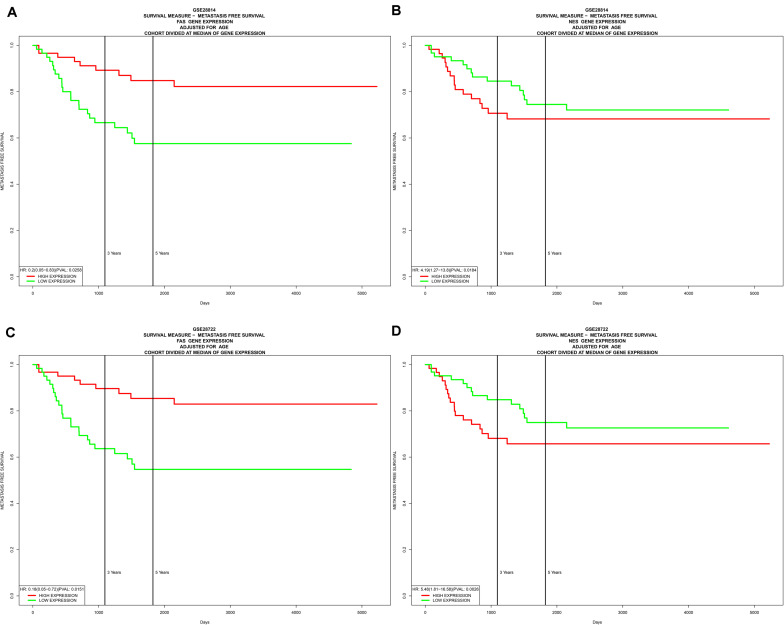
Independent data sets validation of metastasis-free survival (MFS) by GSE28814 and GSE28722. In the two independent data sets, both FAS (GSE28814: *P* = 0.026; GSE28722: *P* = 0.015) **(A,C)** and NES (GSE28814: *P* = 0.018; GSE28722: *P* = 0.003) **(B,D)** had significant metastasis-free survival (MFS) with patients.

**FIGURE 12:**
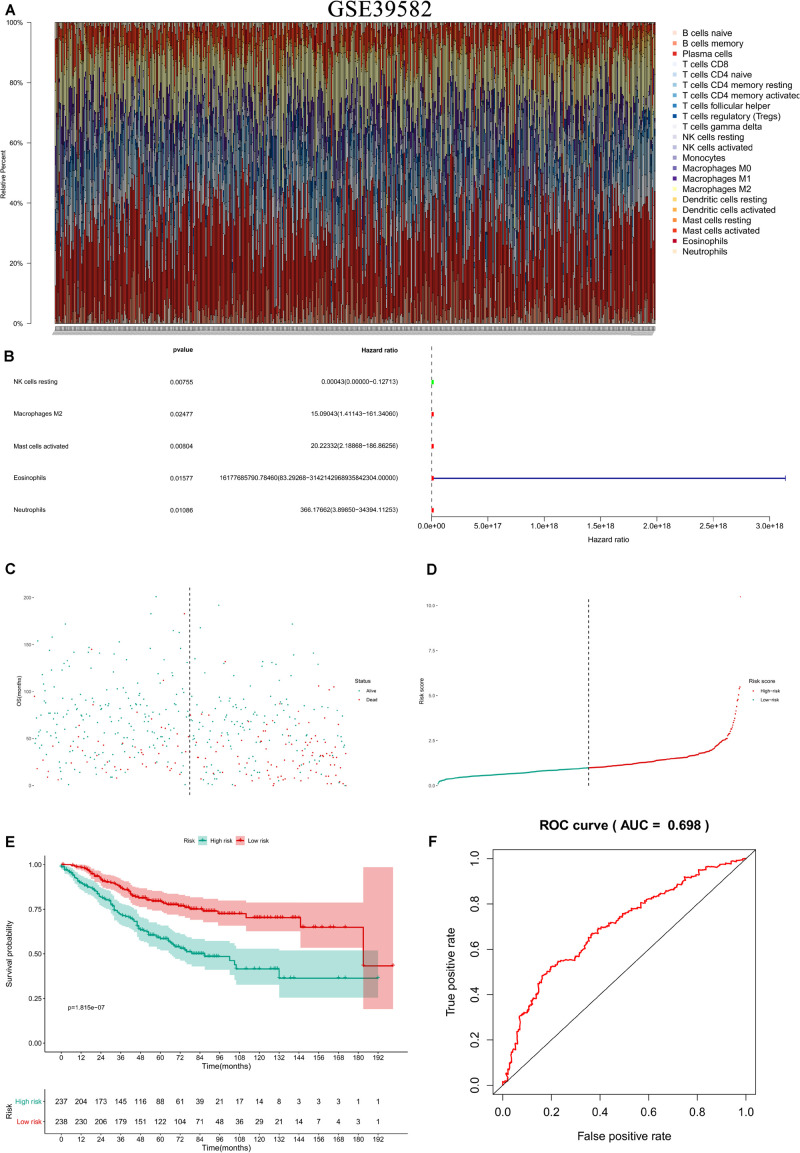
Independent data sets validation of CIBERSORT by GSE39582. GSE39582 with 585 COAD samples was used to validation of CIBERSORT **(A)**. The results suggested that Macrophages M2 had significantly prognostic value for COAD **(B)**. Furthermore, the risk score for each COAD patient was calculated by the formula of multivariate Cox regression model. The distribution of risk score among all COAD patients were shown by the risk line and risk scatterplot **(C,D)**. Kaplan-Meier survival curve suggested risk score had prognostic value for COAD patients (**E**, *P* < 0.001). And the ROC curve (AUC = 0.698) illustrated acceptable discrimination of the multivariate Cox regression model **(F)**.

In the two independent data sets, both FAS (GSE28814: *P* = 0.026; GSE28722: *P* = 0.015) and NES (GSE28814: *P* = 0.018; GSE28722: *P* = 0.003) had significant metastasis-free survival (MFS) with patients (Loboda et al., 2011; Marisa et al., 2013; Figure 11). Additionally, GSE39582 with 585 COAD samples was used to validation of CIBERSORT (Marisa et al., 2013; Figure 12A). The results suggested that Macrophages M2 had significantly prognostic value for COAD (Figure 12B). Furthermore, the risk score for each COAD patient was calculated by the formula of multivariate Cox regression model. The distribution of risk score among all COAD patients were shown by the risk line and risk scatterplot (Figures 12C–D). Kaplan-Meier survival curve suggested risk score had prognostic value for COAD patients (Figure 12E, *P* < 0.001). And the ROC curve (AUC = 0.698) illustrated acceptable discrimination of the multivariate Cox regression model (Figure 12F).

## Discussion

Metastasis of COAD which causes more than one million new COAD cases and about 700,000 death every year (Haggar and Boushey, 2009) is the most frequent cause of treatment failure (Stein et al., 2009). Besides, metastases could significantly reduce the 5-year survival rates for patients (O’connell et al., 2004). Various genetic and molecular biomarkers like protein-coding genes and non-coding genes are used in diagnosis and therapeutic targets in the metastasis of COAD (Asghar et al., 2010; Bokemeyer et al., 2011; Cheng et al., 2011; Toiyama et al., 2012; Zlobec, 2013; Hur, 2015). Among them, metastasis-specific ceRNAs (competitive endogenous RNAs) and tumor-infiltrating immune cells caught our eyes. Nevertheless, there are few studies concentrating on them before.

Our study found differently expressed ceRNAs and marked tumor-infiltrating immune cells between COAD metastasis group and non-metastasis group firstly. Then, two prediction nomograms were drawn based on the selected ceRNAs and tumor-infiltrating immune cells. And AUC values of the two nomograms might be used to estimate the clinical diagnosis of COAD metastasis. In addition, we found that metastasis-related ceRNAs of hsa-miR-125b-5p and FAS and immune cells of Macrophages M0 and T cells follicular helper could predict prognosis effectively. At last, the correlation analysis showed that hsa-miR-125b-5p was associated with macrophages M0 (*R* = 0.170, *P* < 0.001) and T cells follicular helper (*R* = −0.200, *P* < 0.001) significantly. Meanwhile, FAS was associated with macrophages M0 (*R* = −0.370, *P* < 0.001) significantly. We could deduce that the three pairs and their relevant mechanisms would play important roles in the prediction and cure of COAD metastasis.

Hsa-miR-125b-5p is a microRNA whose family has already became biomarkers for cancer diagnosis, treatment and prognosis (Yin et al., 2015). It is also expressed in the patients with rheumatic mitral valve disease and acute ischemic stroke (Liu et al., 2014; Tiedt et al., 2017). Meanwhile, a study based on a Chinese patient population showed that hsa-miR-125b-5p was screened out for the early diagnosis of COAD (Wang et al., 2016) and another study concerned about the function of hsa-miR-125b-5p in COAD carcinogenesis independent from the MTUS1 (Ozcan et al., 2016). However, there are few studies about hsa-miR-125b-5p and COAD metastasis. Macrophages which play a significant role in the metastatic processes are myeloid cells and tumor-infiltrating immune cells (Kim et al., 2009; Kitamura et al., 2015). Infiltration of macrophages in the tumor stroma are negative prognostic factors in COAD (Waniczek et al., 2017). It has been confirmed that the α7 nicotinic acetylcholine receptor of tumor-associated macrophages could inhibit the metastasis of COAD through the JAK2/STAT3 signaling pathway (Fei et al., 2017). What’s more, macrophages are critical factors in the process of liver metastasis of COAD (Zhou et al., 2010; Imano et al., 2011; Cui et al., 2013; Kee et al., 2014). In our present study, we found hsa-miR-125b-5p from ceRNA network, macrophages M0 which were selected from CIEBERSORT analysis and the co-expression relationship of hsa-miR-125b-5p and macrophages M0. The correlation analysis result went along with the former studies that revealed some regulated connection between macrophages M0 and hsa-miR-125b-5p (Diao et al., 2017; Luo et al., 2019). T cells follicular helper from CD4+T cell subset help B cells induce antibody response (Crotty, 2011). One study suggested the involvement of T cells follicular helper in Crohn’s disease-associated COAD (Wang et al., 2014). In COAD, T cells follicular helper improve the effector functions of CD8+ T cells through IL-21supplement, which is downregulated by PD-1/PD-L1-mediated suppression (Shi et al., 2018). Furthermore, T cells follicular helper and B cells combined with VEGF-A enhancement promote neovascularization in peritoneal carcinomatosis which is a terminal evolution from primary COAD (Seebauer et al., 2016). Nevertheless, there are few researches before about the connections of follicular helper T cells and hsa-miR-125b-5p. Thus, we inferred that hsa-miR-125b-5p, as a miRNA, might associated to macrophages M0 and T cells follicular helper and took active parts in the metastasis of COAD.

FAS, a cell surface receptor from the tumor necrosis factor (TNF) receptor superfamily, mediates the apoptosis process (Suda et al., 1993; Dhein et al., 1995). One study based on the analysis of mRNA level showed that the progression of COAD is associated with significantly increased expression of FAS receptor (FASR) and/or FAS ligand (FASL) (Szarynska et al., 2017). FASL and FASR could become good therapeutic targets for COAD (Pryczynicz et al., 2010) and the expression of FAS ligand was found as an early event in the genesis of COAD (Shimoyama et al., 2001). Furthermore, FAS signaling promotes COAD metastasis through inducing epithelial-mesenchymal transition (Chen et al., 2017). The significant change of sFAS/sFASL ratio from metastatic COAD treatment response might be an indicator of chemosensitivity (Yildiz et al., 2010). FAS resisted the apoptosis of metastatic COAD cells by participating in the adjustment of its gene products (Huerta et al., 2007). FASL was found to play a vital role in mediating some physiological functions of macrophages (Kiener et al., 1997). In addition, Sugita et al. found that macrophage activated by FASL+ was an important host defense mechanism involved in the resistance of cancer cell spreading in COAD (Sugita et al., 2002). But after systematic literature review, no explicit reports on FAS associated with macrophages M0 were found in the metastasis of COAD. The definite correlations of FAS and macrophages M0 in COAD metastasis remains unknown and our study will help us learn more about them.

In our study, some restrictions which should be acknowledged do inevitably exist. First of all, the amount of data we collect from publicly available databases is indeed limited, which means that the clinical pathology parameters analyzed in our study were not completely all-inclusive and might bring about potential errors or partialities. In addition, we didn’t consider the heterogeneity of the immune microenvironment which is allied to the immune infiltration location. Last but not the least, the biggest problem in this study is the lack of experiments on the direct interaction mechanisms of ceRNA and cellular communication [e.g., MS2-TRAP (MS2- tagged RNA affinity purification) and RIP (RNA Immunoprecipitation)]. However, in order to diminish the bias, multiple databases were also utilized to uncover the expression of key biomarkers at gene expression, protein expression, tissue and cellular levels and it illustrated that the key biomarkers were regulated upward in normal COAD tissues and cell lines significantly and normal colon tissue didn’t express these proteins (Supplementary Figures S2–S8). Since the scientific hypothesis of this study was inspired by some basic experimental studies, which reported that exosomes secreted by tumor cells contain ceRNAs, exosomes act upon immune cells, and ultimately mediate phenotypes (Li S. et al., 2018; Li et al., 2018a, b; Zhang et al., 2019; Zhao Y. et al., 2019), this study also provided valuable data for further research, in which we would explore the straight molecular mechanisms of the ceRNAs distinctive to metastatic COAD and the intercellular communication between COAD cells and macrophages.

## Conclusion

Basing on the ceRNA network and tumor-infiltrating immune cells we studied, we constructed two nomograms, in order to predict survival and metastasis of COAD patients. The utility was further attested with their high AUC values. What’s more, the prediction nomograms might provide a good deal of useful comprehensive clinical information for doctors, so as to improve individual management for COAD patients. Moreover, our study inferred that the mechanism of hsa-miR-125b-5p. FAS and Macrophages M0 might be important in COAD prognosis and while T cells follicular helper, Macrophages M0 and hsa-miR-125b-5p, Macrophages M0 and FAS played significant parts of the distance metastasis of COAD.

## Data Availability Statement

Publicly available datasets were analyzed in this study. This data can be found here: The code and datasets generated and/or analyzed during the current study are available in the Supplementary Material and the raw data of this study are available in TCGA-COAD program (https://portal.gdc.cancer.gov), Gene Expression Omnibus (GEO) (https://www.ncbi.nlm.nih.gov/geo/) (Accessions: GSE39582, GSE28814, and GSE28722).

## Ethics Statement

The studies involving human participants were reviewed and approved by the Ethics Committee of the Shanghai Tenth People’s Hospital affiliated to Tongji University. The patients/participants provided their written informed consent to participate in this study.

## Author Contributions

ZC, RH, WF, TM, ZH, QW, and HQ: conception/design and data analysis and interpretation. TM, ZH, QW, and HQ: provision of study material. ZC, RH, WF, JL, GJ, JH, WS, HY, WW, TM, ZH, QW, and HQ: collection and/or assembly of data, manuscript writing, and final approval of manuscript. All authors contributed to the article and approved the submitted version.

## Conflict of Interest

The authors declare that the research was conducted in the absence of any commercial or financial relationships that could be construed as a potential conflict of interest.

## Footnotes

1 https://tcga-data.nci.nih.gov/tcga/

2 http://mirtarbase.mbc.nctu.edu.tw/

3 http://carolina.imis.athena-innovation.gr/diana_tools/web/index.php?r=lncbasev2/index-experimental

## Supplementary Material

FIGURE S1 The result of the Kaplar-Meier survival curve revealed that distance metastasis was indeed a prominent risk factor for poor prognosis of COADs (*P* < 0.001).

FIGURE S2 FAS (Median rank 347.0, *P* = 4.98E-6) (**A,B**) was lowly expressed in primary COAD compared to normal colon and some gene symbols of T cells follicular helper such as CD3E (Median rank 109.0, P = 1.81E-6) **(C,D)**, MME (Median rank 6102.5, *P* = 0.027) **(E,F)**, CXCL13 (Median rank 406.0, *P* = 1.41E-8) **(G,H)**, and BCL6 (Median rank 6472.0, *P* = 0.014) **(I,J)** showed obvious difference in the Oncomine.

FIGURE S3 The correlation of FAS, CXCL13 (*P* < 0.001, *R* = 0.400) **(A,B)** and BCL6 (*P* < 0.001, *R* = 0.340) **(C,D)** was significantly different between COAD and normal colon in GEPIA at the transcriptome level.

FIGURE S4 The validation results of LinkedOmics database. In LinkedOmics database, Pearson-correlation analysis also drew significant results between EGFR and CXCL13 (*P* = 4.536E-5), CD3D (*P* = 1.511E-13) and CD3E (*P* = 4.536E-5) **(A–C)**. Besides, FAS (*P* = 4.623E-6) and hsa-miR-125b-5p (*P* = 1.102E-9) were significantly related to tumor purity and tumor stage of COAD **(D–I)**. What’s more, the volcano plot and heatmaps show top 100 proteins (50 positive and 50 negative) with significant correlations with the gene expression level of FAS based on the reverse-phase protein arrays (RPPA) and RNA-seq data **(J–L)**.

FIGURE S5 The results of data mining of The Human Protein Atlas indicated the detection of protein FAS **(A)**, CD3G **(B)** and MME **(C)** were significantly different in COAD and normal colon.

FIGURE S6 The results of String database showed the protein-protein interaction network of FAS, CD3D, and CD3E.

FIGURE S7 The base sequences and duplex structures of miR-320a **(A)** and miR-125b-5p **(B)** available from the MirTarBase.

FIGURE S8 The results of data mining of exoRBase. The scientific hypothesis of this study was inspired by some basic experimental studies, which reported that exosomes secreted by tumor cells contain ceRNAs, exosomes act on immune cells, and ultimately mediate phenotypes. Therefore, the expression levels of key lncRNA (LINC01116) and mRNAs (FAS and NES) were validated in the RNA-seq data of exosomes by exoRBase.

TABLE S1 The baseline information of 459 colon carcinoma patients which were available from the TCGA. SD, Standard deviation.

## Abbreviations

AUC: area under curve
CCLE: cancer cell line encyclopedia
ceRNAs: competing endogenous RNAs
CIBERSORT: cell type identification by estimating relative subsets of RNA transcripts
COAD: colon adenocarcinomas
FPKM: fragments per kilobase of exon per million reads mapped
GEPIA: gene expression profiling interactive analysis
GTEx: genotype-tissue expression
lncRNAs: long non-coding RNAs
miRNA: microRNA
ROC: receiver operating characteristic curves
TCGA: the cancer genome atlas
TNF: tumor necrosis factor.
