# Supplementary figures and images for: The Construction and Analysis of ceRNA Network and Patterns of Immune Infiltration in Colon Adenocarcinoma Metastasis
Source: Front Cell Dev Biol. 2020 Aug 4;8:688. doi: 10.3389/fcell.2020.00688 (PMC7417319; doi:10.3389/fcell.2020.00688)

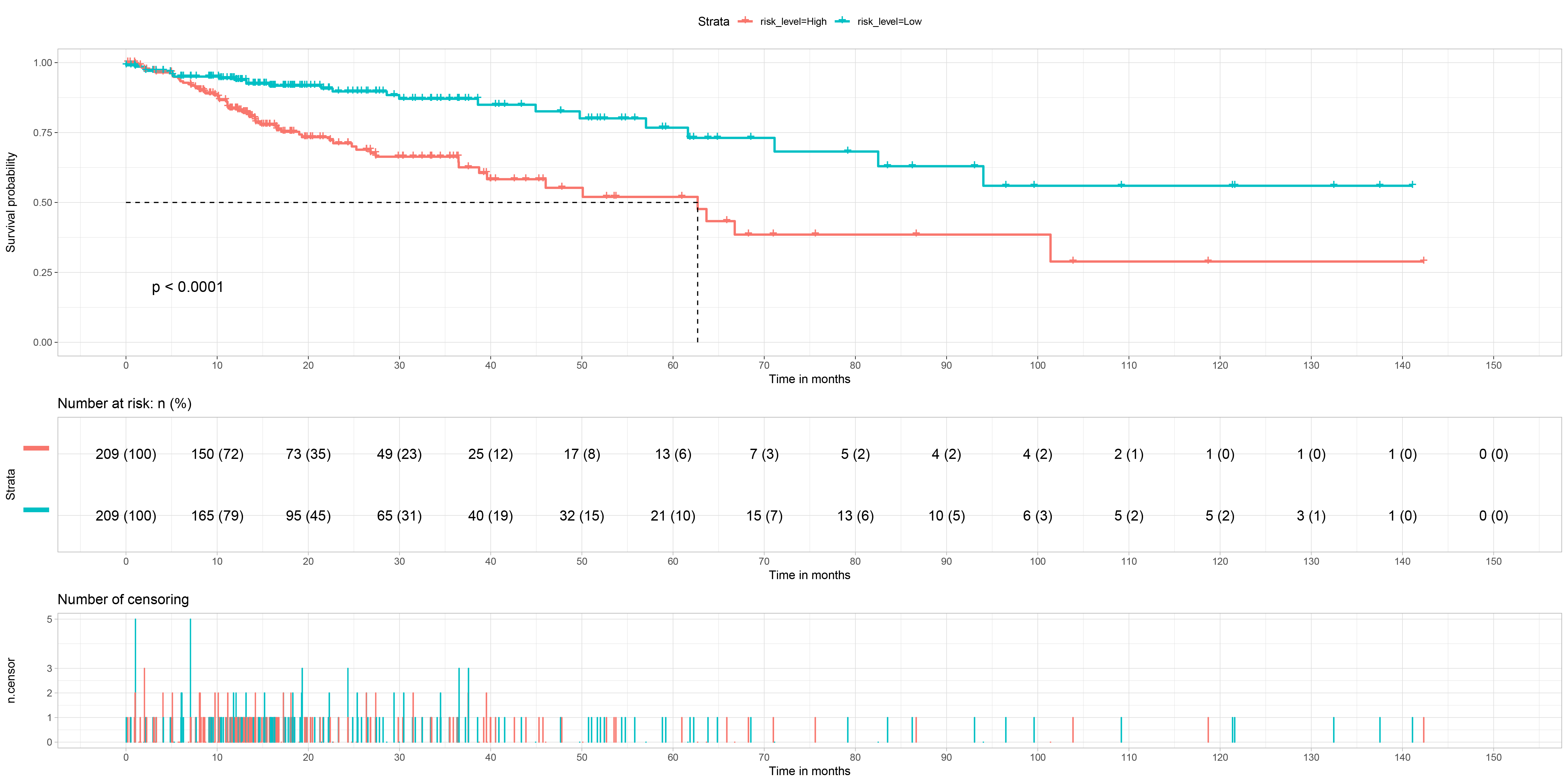

Supplement: FIGURE S1 — The result of the Kaplar-Meier survival curve revealed that distance metastasis was indeed a prominent risk factor for poor prognosis of COADs (P < 0.001). [file Image_1.TIF]

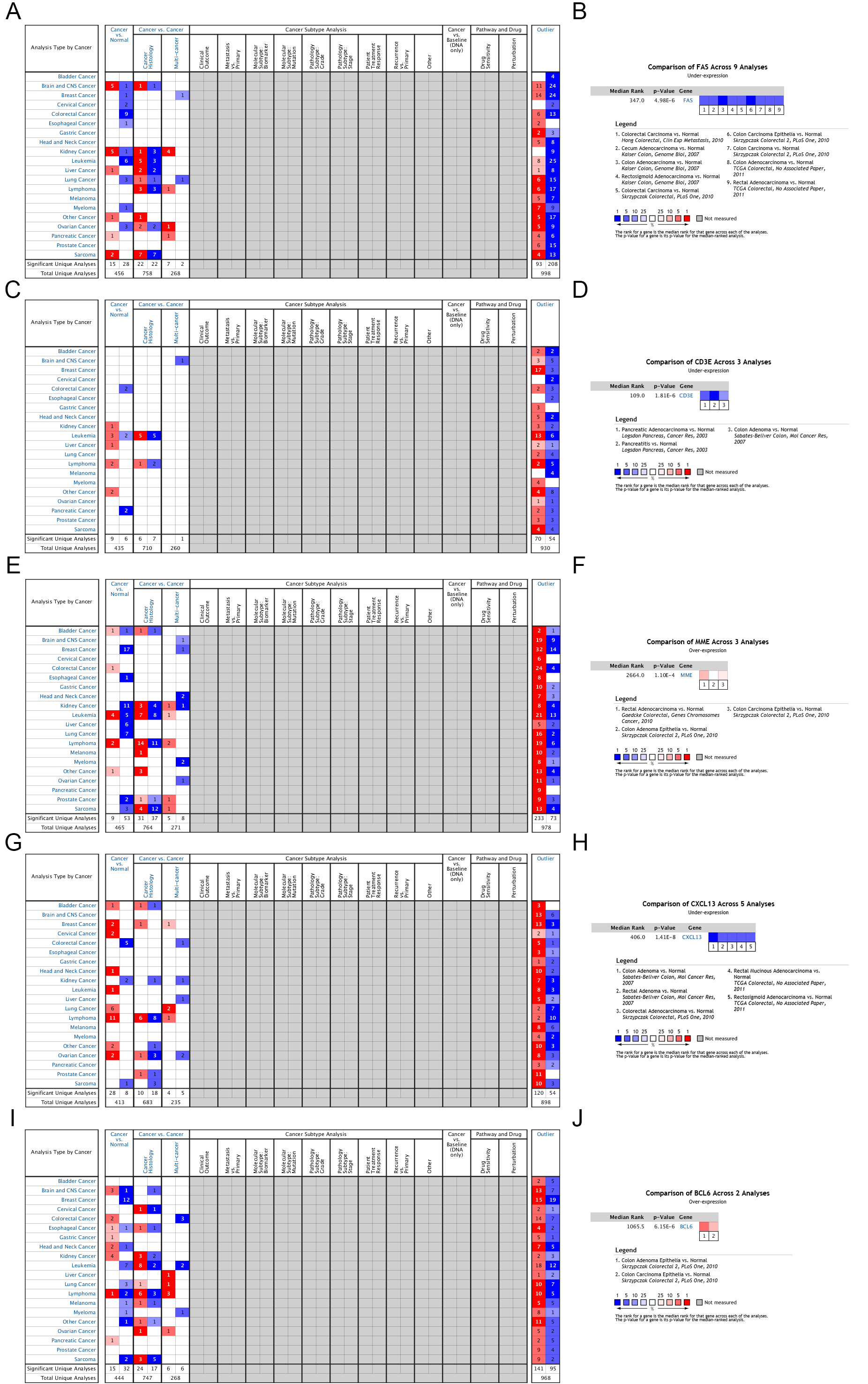

Supplement: FIGURE S2 — FAS (Median rank 347.0, P = 4.98E-6) (A,B) was lowly expressed in primary COAD compared to normal colon and some gene symbols of T cells follicular helper such as CD3E (Median rank 109.0, P = 1.81E-6) (C,D), MME (Median rank 6102.5, P = 0.027) (E,F), CXCL13 (Median rank 406.0, P = 1.41E-8) (G,H), and BCL6 (Median rank 6472.0, P = 0.014) (I,J) showed obvious difference in the Oncomine. [file Image_2.TIF]

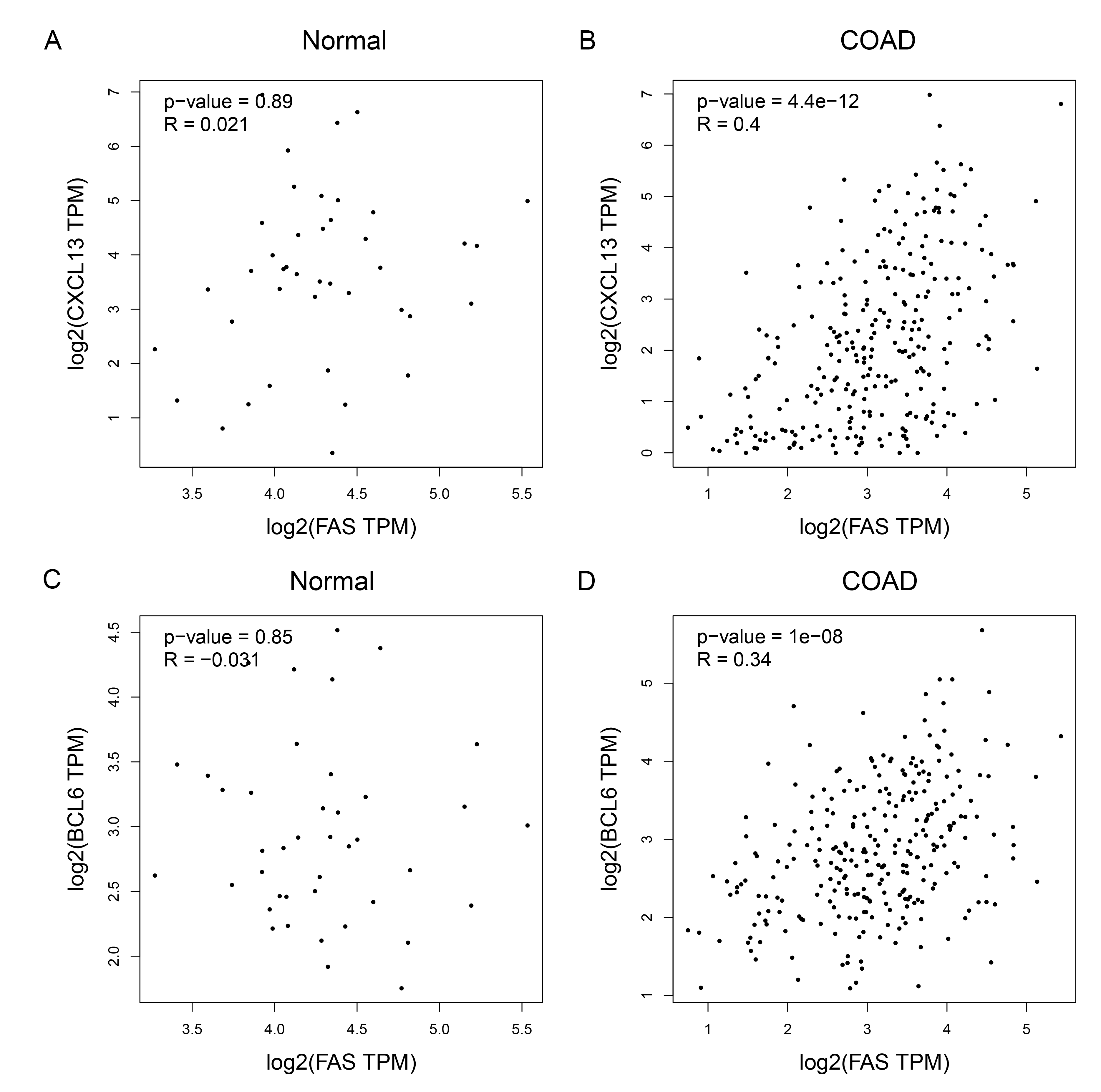

Supplement: FIGURE S3 — The correlation of FAS, CXCL13 (P < 0.001, R = 0.400) (A,B) and BCL6 (P < 0.001, R = 0.340) (C,D) was significantly different between COAD and normal colon in GEPIA at the transcriptome level. [file Image_3.TIF]

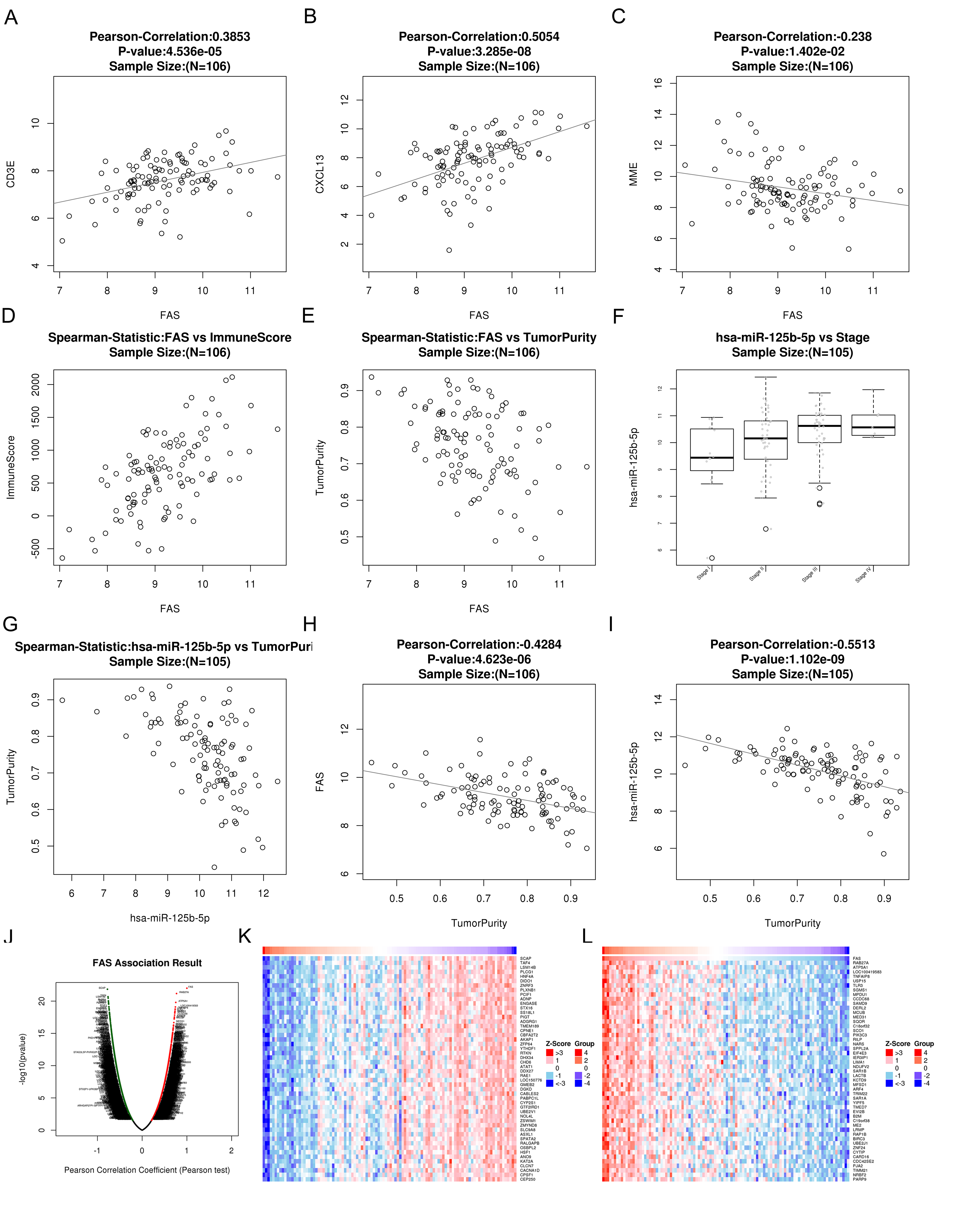

Supplement: FIGURE S4 — The validation results of LinkedOmics database. In LinkedOmics database, Pearson-correlation analysis also drew significant results between EGFR and CXCL13 (P = 4.536E-5), CD3D (P = 1.511E-13) and CD3E (P = 4.536E-5) (A–C). Besides, FAS (P = 4.623E-6) and hsa-miR-125b-5p (P = 1.102E-9) were significantly related to tumor purity and tumor stage of COAD (D–I). What’s more, the volcano plot and heatmaps show top 100 proteins (50 positive and 50 negative) with significant correlations with the gene expression level of FAS based on the reverse-phase protein arrays (RPPA) and RNA-seq data (J–L). [file Image_4.TIF]

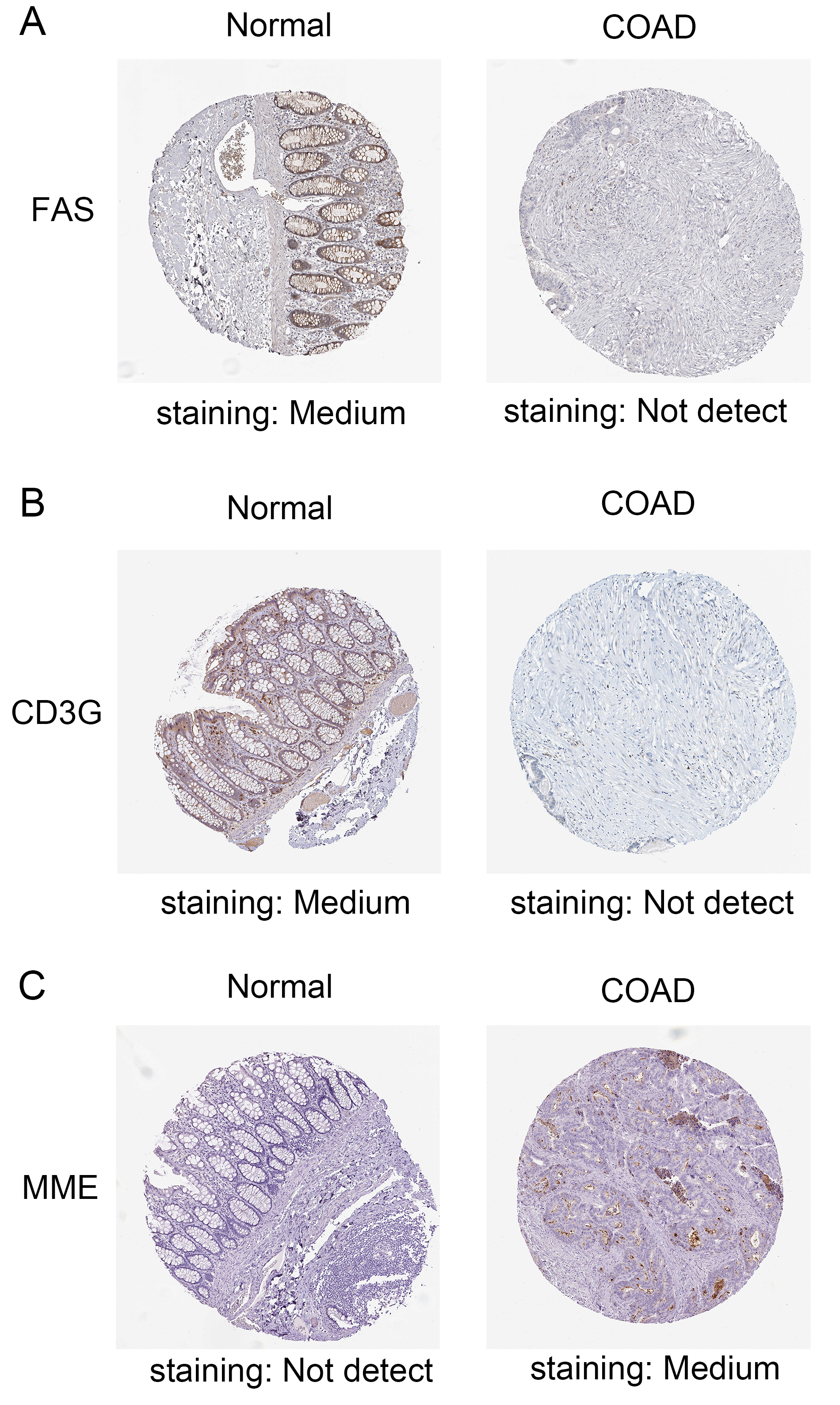

Supplement: FIGURE S5 — The results of data mining of The Human Protein Atlas indicated the detection of protein FAS (A), CD3G (B) and MME (C) were significantly different in COAD and normal colon. [file Image_5.TIF]

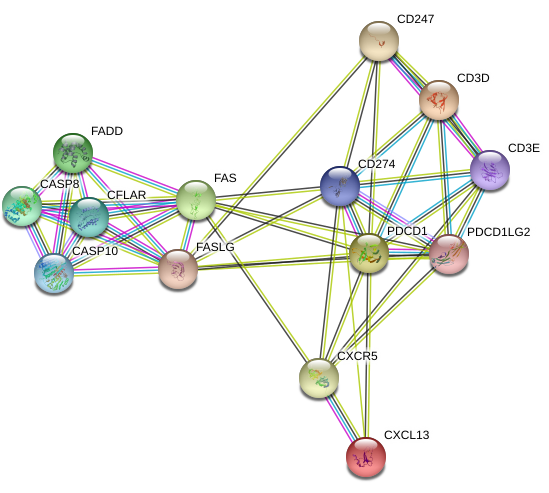

Supplement: FIGURE S6 — The results of String database showed the protein-protein interaction network of FAS, CD3D, and CD3E. [file Image_6.TIF]

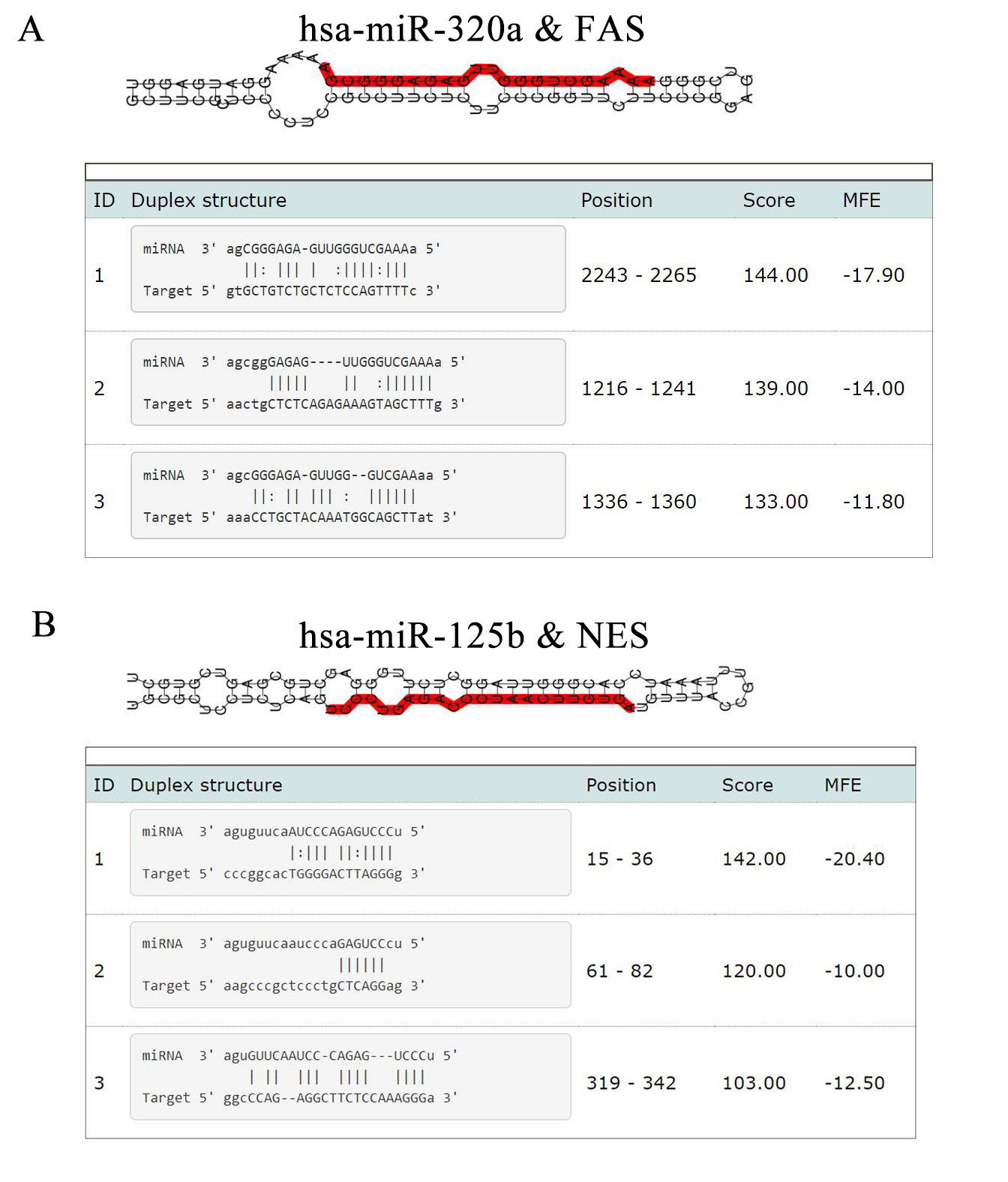

Supplement: FIGURE S7 — The base sequences and duplex structures of miR-320a (A) and miR-125b-5p (B) available from the MirTarBase. [file Image_7.TIF]

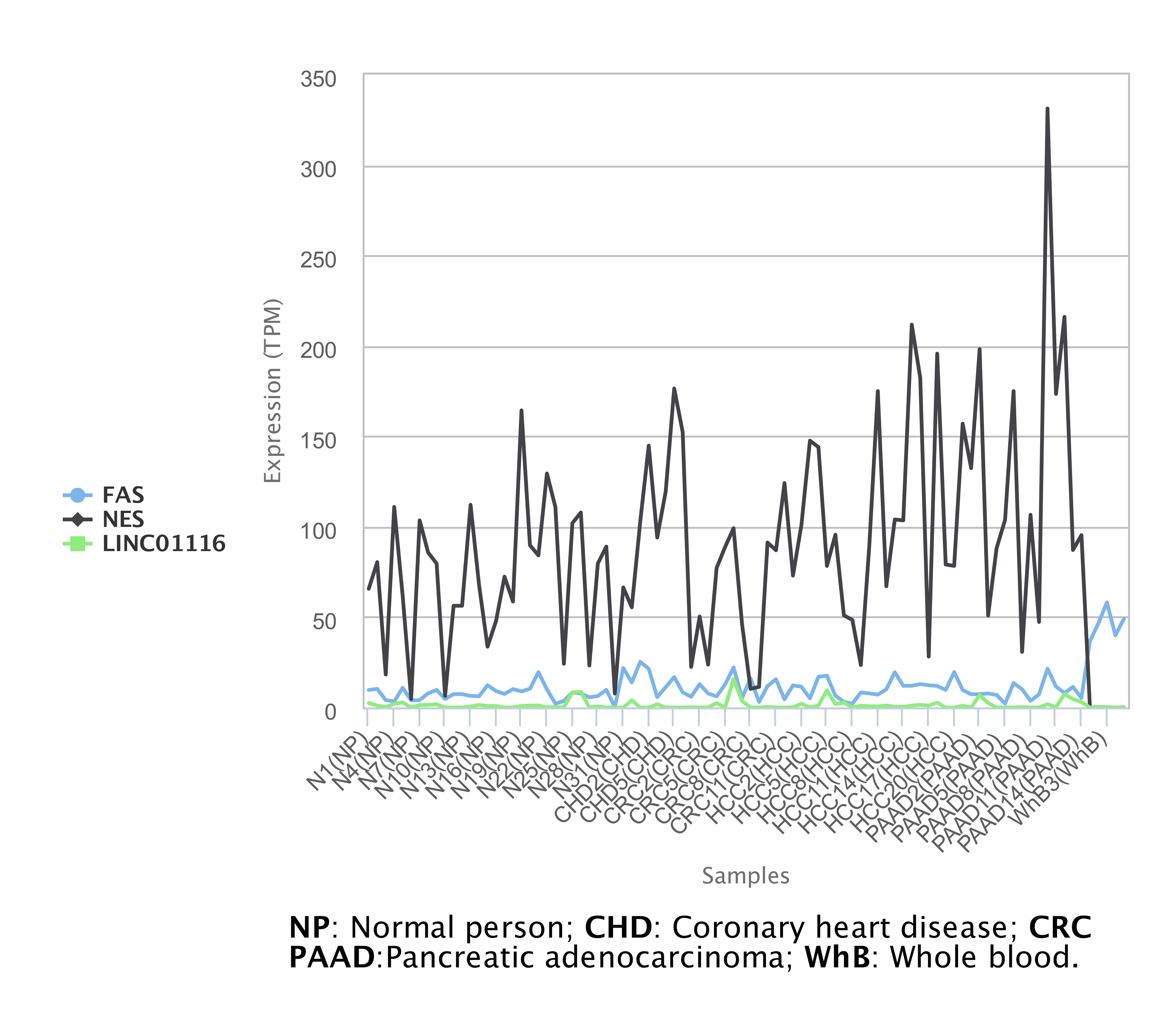

Supplement: FIGURE S8 — The results of data mining of exoRBase. The scientific hypothesis of this study was inspired by some basic experimental studies, which reported that exosomes secreted by tumor cells contain ceRNAs, exosomes act on immune cells, and ultimately mediate phenotypes. Therefore, the expression levels of key lncRNA (LINC01116) and mRNAs (FAS and NES) were validated in the RNA-seq data of exosomes by exoRBase. [file Image_8.TIF]
