# Supplementary material for: The Construction and Analysis of ceRNA Network and Patterns of Immune Infiltration in Colon Adenocarcinoma Metastasis
Source: Front Cell Dev Biol. 2020 Aug 4;8:688. doi: 10.3389/fcell.2020.00688 (PMC7417319; doi:10.3389/fcell.2020.00688)
Supplement: TABLE S1 — The baseline information of 459 colon carcinoma patients which were available from the TCGA. SD, Standard deviation. [file Table_1.docx]

**Table S1.** Baseline information of 459 patients diagnosed with colon carcinoma.

| Variables | Total Patients (N = 459) |
| --- | --- |
| **Age, years** |  |
| Mean ± SD | 66.92 ± 13.08 |
| Median (Range) | 68.00 (31 -90) |
| **Gender** |  |
| Female | 216 (56.86%) |
| Male | 243 (52.94%) |
| **Race** |  |
| Asian | 11 (2.40%) |
| Black or African American | 59 (12.85%) |
| White | 214 (46.62%) |
| Unknown | 175 (38.13%) |
| **Vital status** |  |
| Dead | 102（22.22%） |
| Alive | 357（77.78%） |
| **Days to death** |  |
| Less than 500 days | 56（12.20%） |
| More than 500 days | 39（8.50%） |
| Unknown | 364（79.30%） |
| **Days to last follow up** |  |
| Less than 500 days | 127（27.66%） |
| More than500 days | 256（55.77%） |
| Unknown | 76（16.57%） |
| **New tumor event** |  |
| Yes | 91（19.83%） |
| Unknown | 368（80.17%） |
| **Days to new tumor event after initial treatment** |  |
| Less than 500 days | 50（10.89%） |
| More than 500 days | 38（8.28%） |
| Unknown | 371（80.83%） |
| **New neoplasm event type** |  |
| Locoregional Disease | 9（1.96%） |
| Metastasis | 9（1.96%） |
| Unknown | 441（96.08%） |
